# Factors associated with re-initiation of antidepressant treatment following discontinuation during pregnancy: a register-based cohort study

**DOI:** 10.1007/s00737-020-01050-y

**Published:** 2020-07-06

**Authors:** Anna Wikman, Alkistis Skalkidou, Anna-Karin Wikström, Erik Lampa, Michael S. Kramer, Eu-Leong Yong, Charlotte Skoglund, Neill Epperson, Inger Sundström-Poromaa

**Affiliations:** 1 Department of Women’ and Children’s Health, Uppsala University, Uppsala, Sweden; 2 Uppsala Clinical Research Centre, Uppsala University, Uppsala, Sweden; 3 Departments of Epidemiology, Biostatistics & Occupational Health and of Pediatrics, McGill University Faculty of Medicine, Montreal, Quebec, Canada; 4 Department of Obstetrics and Gynaecology, National University Hospital, National University of Singapore, Singapore; 5 Department of Clinical Neuroscience, Karolinska Institutet, Stockholm, Sweden; 6 Department of Psychiatry, University of Colorado School of Medicine, Aurora, Colorado USA

**Keywords:** Mental health, Peripartum depression, Pregnancy, Antidepressants

## Abstract

Antidepressant treatment when facing a pregnancy is an important issue for many women and their physicians. We hypothesized that women with a greater burden of pre-pregnancy psychiatric illness would be more likely to re-initiate antidepressants following discontinuation of treatment during pregnancy. A register-based cohort study was carried out including 38,595 women who gave birth between the 1st of January 2007 and the 31st of December 2014, who had filled a prescription for an antidepressant medication in the year prior to conception. Logistic regressions were used to explore associations between maternal characteristics and antidepressant treatment discontinuation or re-initiation during pregnancy. Most women discontinued antidepressant treatment during pregnancy (*n* = 29,095, 75.4%), of whom nearly 12% (*n* = 3434, 11.8%) re-initiated treatment during pregnancy. In adjusted analyses, parous women (aOR 1.22, 95% CI 1.12–1.33), with high educational level (aOR 1.21, 95% CI 1.08–1.36); born within the EU (excluding Nordic countries, aOR 1.41, 95% CI 1.03–1.92) or a Nordic country (aOR 1.42, 95% CI 1.22–1.65); who more often reported prior hospitalizations due to psychiatric disorders (aOR 1.50, 95% CI 1.10–2.03, for three or more episodes); and had longer duration of pre-pregnancy antidepressant use (aOR 6.10, 95% CI 5.48–6.77, for >2 years antidepressant use), were more likely to re-initiate antidepressants than were women who remained off treatment. Women with a greater burden of pre-pregnancy psychiatric illness were more likely to re-initiate antidepressants. Thus, pre-pregnancy psychiatric history may be particularly important for weighing the risks and benefits of discontinuing antidepressants during pregnancy.

## Introduction

Psychiatric disorders in the perinatal period are common (Howard et al. 2014; Vesga-Lopez et al. 2008), and approximately 12% of pregnant women experience perinatal depressive disorder (Woody et al. 2017). Owing to adverse outcomes associated with perinatal depression for both mother and offspring, detection and appropriate treatment of depression is of great importance (Grigoriadis et al. 2013; Grote et al. 2010; Jarde et al. 2016). Among pregnant women in Europe, approximately 3% are treated with selective serotonin reuptake inhibitors (SSRIs); the corresponding rate in North America is 4–10% (El Marroun et al. 2012; Huybrechts et al. 2013; Kieler 2010; Petersen et al. 2018).

Antidepressant treatment during pregnancy remains under debate as evidence on fetal and child outcomes is still inconclusive. Observational research on the use of antidepressant medications in pregnancy suggests these medications are associated with increased risk of preterm delivery and low birth weight (Eke et al. 2016). However, depression itself has also been associated with increased risk of those same outcomes (Jarde et al. 2016) as well as impaired childhood cognitive development (Liu et al. 2017a). Depression during pregnancy has also been found to increase overall risk of psychiatric disorders in offspring (Liu et al. 2017b). Others have found risk of low birth weight and related outcomes not to differ between antidepressant-treated pregnant women and untreated depressed women (Mitchell and Goodman 2018). One recent systematic review suggests antidepressant exposure during pregnancy is associated with an increased risk of lower gestational age and preterm birth, but not low birthweight or being small for gestational age compared with untreated depression, and conflicting evidence emerged regarding neurodevelopment in offspring (Fitton et al. 2019). Overall, the evidence suggests a generally small risk of congenital malformations as a consequence of treatment with selective serotonin reuptake inhibitors (SSRIs) during early pregnancy (Gao et al. 2018).

The decision to treat or not to treat depression with antidepressant medications is fraught with difficulty, weighing the risks of untreated maternal depression against potential adverse effects of antidepressant exposure (National Institute for Health and Care Excellence [NICE] 2018; Ross et al. 2013). Thus, both women and clinicians lack guidance in facing the complex decision regarding initiation, continuation, or discontinuation of antidepressants during pregnancy. Studies show around 40–50% of women discontinue their antidepressant treatment, either before or during pregnancy (Charlton et al. 2015; Hanley and Mintzes 2014; Molenaar et al. 2020a).

Few studies have examined the risks associated with discontinuation of antidepressant medication during pregnancy, and the risk of relapse is extremely variable between the ones that have addressed the issue. One previous study found that 68% of pregnant women who discontinued antidepressant medication use during pregnancy had a relapse of major depression during pregnancy, compared with 26% of those who continued their treatment. The risk of relapse was particularly high for women with multiple previous depressive episodes. Among women who reduced the dose or discontinued their antidepressant medication, 61% reinitiated treatment during pregnancy (Cohen et al. 2006). Similarly, results from a small prospective study aiming to identify factors associated with SSRI discontinuation during pregnancy found more than half of women discontinued treatment upon confirmation of pregnancy. Of these, nearly 60% reinitiated treatment (Roca et al. 2013). Others report low recurrence rates and no significant effect of antidepressant discontinuation on recurrence risk (Yonkers et al. 2011). One previous large epidemiological study found women who continued antidepressant treatment during pregnancy were more likely to have a depression inpatient stay than were women who had discontinued treatment (Swanson et al. 2015). These results might suggest that women who continue antidepressant medication are also those with a more severe course, and therefore are at higher risk of inpatient treatment, despite continuing treatment.

Based on these discrepancies, we aimed to provide better estimates of the likelihood of antidepressant re-initiation following discontinuation of antidepressant treatment during pregnancy in a large population and register-based study. We explored the maternal sociodemographic and clinical characteristics of women who continued and those who discontinued antidepressants, and between those who remained off compared with those who re-initiated antidepressant treatment during pregnancy. We hypothesized that women with a greater burden of pre-pregnancy psychiatric illness would be more likely to re-initiate antidepressant treatment during pregnancy.

## Methods

### Data sources

A nationwide, population-based cohort study was carried out using data from five national longitudinal population-based health registers in Sweden: the Swedish Medical Birth Register, the Patient Register and the Prescribed Drug Register, held by the Swedish National Board of Health and Welfare. Data from the Education Register and the Total Population Register were obtained from Statistics Sweden. The unique personal identity number assigned to all Swedish residents at birth or immigration was used to link information in the registers (Ludvigsson et al. 2009).

The Swedish Medical Birth Register covers 98% of all births in Sweden and includes prospectively collected clinical variables, demographic data, information on reproductive history, and complications that occur during pregnancy, delivery, and the neonatal period (Axelsson 2003). The Patient Register includes information on all inpatient care and outpatient specialist services, including visits to psychiatrists, with all procedures and diagnoses documented using International Classification of Diseases (ICD) diagnostic codes, dates of visits, and hospital admissions for each individual. The register has full nationwide coverage of inpatient care since 1987, and complete coverage of specialist outpatient care since 2001 (Ludvigsson et al. 2011). The Swedish Prescribed Drug Register was introduced in July 2005 and currently includes a record of all medications prescribed and dispensed in Sweden by Anatomical Therapeutic Chemical Classification (ATC) codes and date of dispensing (Wettermark et al. 2007). Ethical approval was granted by the regional ethical review board in Uppsala, Sweden (DNR 2017/031).

### Study population

**Fig. 1:**
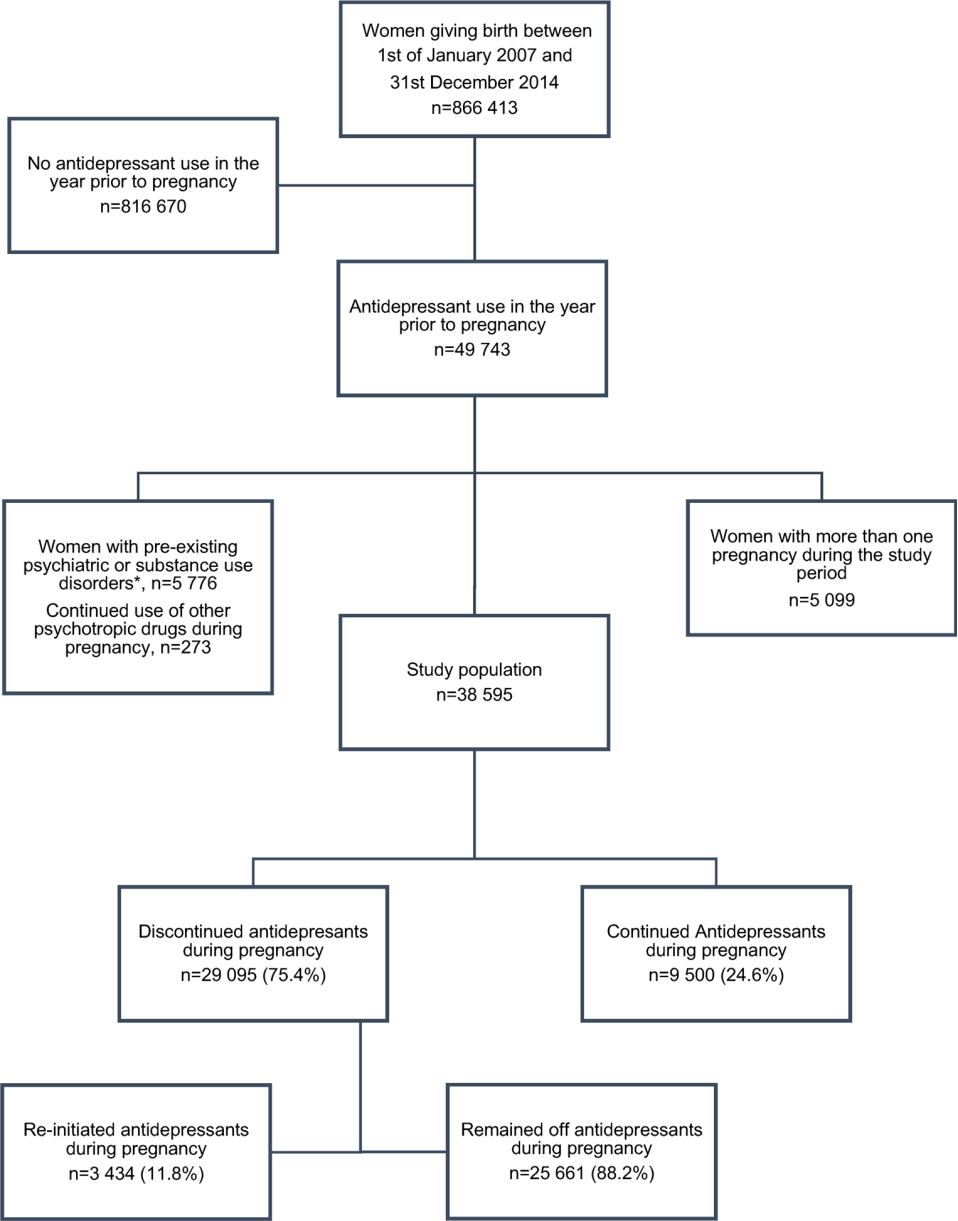
Flowchart of study population

We included all women giving birth between 1 January 2007 and 31 December 2014 who were dispensed an antidepressant drug (ATC code N06A) in the year prior to estimated date of conception. Estimated date of conception was calculated based on information from the Medical Birth Register. As the exact date of birth was not included in the application for data, the year and month of birth were used to estimate date of conception by assuming all women had given birth on the 15th of their month of delivery. The Medical Birth Register also contains information on gestational age at parturition, based on ultrasound dating of pregnancy performed in the early second trimester. Thus, we calculated date of conception as [estimated date of birth − gestational age in days]. Women with more than one pregnancy (any second or subsequent pregnancy) during the study period were excluded. To capture women with predominantly depressive and anxiety disorders, we excluded women with pre-pregnancy bipolar disorder (F30-F31), schizophrenia, and psychotic disorders (F20-F29); emotionally unstable personality disorder (F60.3), attention deficit hyperactivity disorder (F90), or substance use disorders (F10–16, F18-F19); and women who filled a prescription for lithium (ATC-code N05AN), neuroleptic drugs (ATC-code N05A), or central stimulant drugs (ATC-code N06BA) during pregnancy. Thus, the study population comprised 38,595 women (Fig. 1).

### Outcomes

In Sweden, drugs are prescribed for a maximum of 1 year at a time, and the prescription has to be filled every 3 months. Women who were dispensed an antidepressant drug in the year prior to conception but who subsequently did not fill a prescription for antidepressants during the first half of pregnancy, i.e., the first 140 days after estimated date of conception, were defined as having discontinued antidepressant treatment during their pregnancy. Among women who discontinued, those who did not fill any subsequent prescription for an antidepressant during pregnancy were considered remaining off treatment, whereas those who filled a prescription for an antidepressant during the second half of pregnancy were defined as having re-initiated antidepressant treatment, indicating relapse. Women who filled at least one prescription for antidepressant drugs during the first half of the index pregnancy were defined as having continued their antidepressant treatment during pregnancy.

### Sociodemographic and clinical predictors

Information on maternal age at delivery, height, weight, smoking habits, and parity was obtained from the Medical Birth Register. Age at delivery was categorized into the following age groups: < 19, 20–29, 30–39, or ≥ 40 years. Body mass index (BMI, calculated as weight (kg)/[height (m)]^2^) was based on weight and height at first antenatal visit and grouped according to the World Health Organization as < 18.5, 18.5–24.99, 25.0–29.99, 30.0–34.99, 35.0–39.99, and ≥ 40.0 kg/m^2^. Daily smoking at the first antenatal visit was recorded as yes, regardless of quantity. Parity was categorized as nulliparous or parous prior to the study pregnancy.

Data on maternal education attainment by 2014 and country of birth were collected from the Higher Education Registry and the Total Population Register, respectively. Maternal education was categorized as < 12 years, upper secondary, or university level education, and maternal country of birth as Nordic countries, European Union (EU, not including the Nordic countries), or outside the EU. Pre-pregnancy psychiatric hospitalization from the Patient Registry from 1997 and onwards was categorized as never, once, twice, or three or more episodes. Pre-pregnancy antidepressant use was categorized as 3 months or less, 3 months to 1 year, 1–2 years, or more than 2 years.

### Statistical analyses

We carried out logistic regression analysis, estimating unadjusted and adjusted odds ratios (OR) and their 95% confidence intervals (CI), to compare sociodemographic and clinical characteristics between women who continued and those who discontinued antidepressants. To investigate whether women with a greater burden of pre-pregnancy psychiatric illness would be more likely to re-initiate antidepressants during pregnancy compared with remaining off treatment, unadjusted and adjusted logistic regression models were used. To ensure independence of the units of analyses, only one birth per woman during the study period was included (first recorded pregnancy in the database).

## Results

During the study period, 38,595 women were prescribed antidepressant medication in the year before their estimated date of conception. On average, 3 out of 4 women (*n* = 29,095, 75.4%) discontinued antidepressant treatment during pregnancy, and only 1 out of 4 women (*n* = 9500, 24.6%) continued. The percentage of women who discontinued antidepressants during pregnancy appeared to decrease over the time period, with 79.1% of women discontinuing in 2007 compared with 74.3% in 2014. The sociodemographic and clinical characteristics of women who discontinued or continued antidepressants are shown in Table 1. Adjusting for all other sociodemographic and clinical characteristics in the model, women who were older, smoked less, and were born outside the Nordic countries were more likely to discontinue antidepressants during pregnancy. No associations were observed with BMI, parity, and education. The burden of pre-pregnancy psychiatric illness was substantially lower among women who discontinued their antidepressant medication during pregnancy. That is, women with a greater number of in-patient psychiatric hospitalizations prior to the index pregnancy and who had used antidepressants for longer were less likely to discontinue antidepressants (Table 1).

**Table 1:** Sociodemographic and clinical characteristics among women who continued or discontinued antidepressant medication during pregnancy, shown as numbers and percentages, and unadjusted and adjusted odds ratios (OR) with 95% confidence intervals (CI) from logistic regression analyses

Characteristics	Continued AD		Discontinued AD		OR (95% CI)	aOR (95% CI)
	*n* = 9500 (24.6%)		*n* = 29,095 (75.4%)			
	*n*	%	*n*	%		
Age						
≤ 19 years	125	1.3	478	1.7	1.19 (0.97–1.45)	0.95 (0.76–1.18)
20–29 years	3649	38.5	11,759	40.4	(ref)	(ref)
30–39 years	5163	54.3	15,343	52.7	*0.92 (0.88–0.97)*	1.05 (1.00–1.11)
≥ 40 years	563	5.9	1515	5.2	*0.84 (0.75–0.93)*	0.97 (0.86–1.09)
Body mass index (kg/m^2^)						
< 18.5	186	1.9	606	2.1	1.04 (0.89–1.24)	0.96 (0.81–1.15)
18.5–24.9	4615	48.6	14,332	49.2	(ref)	(ref)
25.0–29.9	2475	26.1	7447	25.6	0.97 (0.92–1.03)	0.99 (0.93–1.05)
30.0–34.9	1037	10.9	2963	10.2	0.92 (0.85–1.00)	0.96 (0.88–1.04)
35.0–39.9	373	3.9	1114	3.8	0.96 (0.85–1.09)	1.00 (0.88–1.13)
≥ 40.0	162	1.7	424	1.5	0.84 (0.70–1.01)	0.93 (0.77–1.13)
Missing	652	6.9	2209	7.6		
Smoking						
No	7997	84.2	24,421	83.9	(ref)	(ref)
Yes	1130	11.9	3513	12.1	1.02 (0.95–1.09)	0.92 (0.85–1.00)
Missing	373	3.9	1161	4.0		
Parity						
0 children	5171	54.4	14,773	50.8	(ref)	(ref)
≥ 1 children	4329	45.6	14,322	49.2	*1.16 (1.11–1.21)*	0.97 (0.92–1.03)
Education						
< 12 years	2140	22.5	7125	24.5	(ref)	(ref)
Upper secondary	2652	27.9	8552	29.4	0.97 (0.91–1.03)	1.04 (0.97–1.12)
University	4670	49.2	13,304	45.7	*0.86 (0.81–0.91)*	0.99 (0.92–1.06)
Missing	38	0.4	114	0.4		
Maternal country of origin						
Outside EU	844	8.9	3583	12.3	(ref)	(ref)
EU	200	2.1	653	2.2	*0.77 (0.65–0.92)*	0.83 (0.68–1.00)
Nordic countries	8456	89.0	24,859	85.5	*0.69 (0.64–0.75)*	*0.89 (0.81–0.97)*
Pre-pregnancy psychiatric hospitalization^a^						
Never	8336	87.7	26,430	90.8	(ref)	(ref)
Once	794	8.4	1877	6.5	*0.75 (0.68–0.81)*	*0.84 (0.77–0.93)*
Twice	199	2.1	487	1.7	*0.77 (0.65–0.91)*	0.98 (0.82–1.18)
Three or more episodes	171	1.8	301	1.0	*0.55 (0.46–0.67)*	*0.76 (0.62–0.94)*
Pre-pregnancy antidepressant use						
3 months or less	472	4.9	5359	18.4	*2.44 (2.20–2.71)*	*2.47 (2.21–2.75)*
3 months - 1 year	2229	23.5	10,353	35.6	(ref)	(ref)
1–2 years	2068	21.8	5508	18.9	*0.57 (0.54–0.61)*	*0.58 (0.54–0.62)*
> 2 years	4731	49.8	7875	27.1	*0.36 (0.33–0.38)*	*0.36 (0.34–0.39)*

*AD* antidepressant medication, *CI* confidence interval, *EU* European Union, *OR* odds ratio. Significant OR marked in italics. ^a^F09–F95, except F10–16, F18–F31, F60.3, F90

Among the 29,095 women who discontinued antidepressant medication in the first half of pregnancy, 3434 (11.8%) re-initiated antidepressant treatment during the second half of pregnancy. In 2007, 9.0% of women re-initiated treatment, increasing to 13.3% in 2014. Table 2 compares the sociodemographic and clinical characteristics of these women with those who did not re-initiate their antidepressants for the remainder of their pregnancy. Adjusting for all other sociodemographic and clinical characteristics in the model, women who were multiparous, with a high educational level, were born in the Nordic counties or within the EU (not including the Nordic countries), with a greater number of psychiatric hospitalizations prior to the pregnancy were more likely to re-initiate treatment. However, the greatest difference observed between women who remained off and those who re-initiated was in their previous use of antidepressants. Women who had very short treatment times, i.e., 3 months or less, were less likely to re-initiate treatment (aOR 0.27, 95% CI 0.21–0.35) than were women who had used antidepressants for 3–12 months, whereas those who had used antidepressants for more than 2 years were far more likely to re-initiate treatment (aOR 6.10, 95% CI 5.48–6.77).

**Table 2:** Sociodemographic and clinical characteristics of 29,095 women who discontinued antidepressant medication during pregnancy who either remained off antidepressant medication or who re-initiated antidepressant medication during pregnancy, shown as numbers and percentages, and unadjusted and adjusted odds ratios (OR) with 95% confidence intervals (CI) from logistic regression analyses

Characteristics	Remained off AD		Re-initiated AD		OR (95% CI)	aOR (95% CI)
	*n* = 25,661 (88.2%)		*n* = 3434 (11.8%)			
	*n*	%	*n*	%		
Age						
≤ 19 years	441	1.7	37	1.1	0.73 (0.52–1.03)	1.16 (0.80–1.71)
20–29 years	10,546	41.1	1213	35.3	(ref)	(ref)
30–39 years	13,378	52.1	1965	57.2	*1.28 (1.18–1.38)*	0.96 (0.88–1.06)
≥ 40 years	1296	5.1	219	6.4	*1.47 (1.26–1.72)*	1.12 (0.95–1.34)
Body mass index (kg/m^2^)						
< 18.5	536	2.1	70	2.0	1.00 (0.78–1.29)	1.21 (0.92–1.60)
18.5–24.9	12,681	49.4	1651	48.1	(ref)	(ref)
25.0–29.9	6531	25.4	916	26.7	1.08 (0.99–1.17)	1.06 (0.97–1.17)
30.0–34.9	2593	10.1	370	10.8	1.10 (0.97–1.24)	1.04 (0.91–1.18)
35.0–39.9	966	3.8	148	4.3	1.18 (0.98–1.41)	1.14 (0.94–1.38)
≥ 40.0	365	1.4	59	1.7	1.24 (0.94–1.64)	1.10 (0.82–1.48)
Missing	1989	7.8	220	6.4		
Smoking						
No	21,440	83.5	2981	86.8	(ref)	(ref)
Yes	3173	12.4	340	9.9	*0.77 (0.69–0.87)*	0.94 (0.82–1.07)
Missing	1048	4.1	113	3.3		
Parity						
0 children	12,957	50.5	1816	52.9	(ref)	(ref)
≥ 1 children	12,704	49.5	1618	47.1	*0.91 (0.85–0.98)*	*1.22 (1.12–1.33)*
Education						
<12 years	6460	25.2	665	19.4	(ref)	(ref)
Upper secondary	7600	29.6	952	27.7	*1.22 (1.10–1.35)*	1.10 (0.97–1.23)
University	11,496	44.8	1808	52.6	*1.53 (1.39–1.68)*	*1.20 (1.07–1.34)*
Missing	105	0.4	9	0.3		
Maternal country of origin						
Outside EU	3346	13.0	237	6.9	(ref)	(ref)
EU	587	2.3	66	1.9	*1.59 (1.19–2.11)*	*1.41 (1.03–1.92)*
Nordic countries	21,728	84.7	3131	91.2	*2.03 (1.77–2.33)*	*1.42 (1.22–1.65)*
Pre-pregnancy psychiatric hospitalization^a^						
Never	23,380	91.1	3050	88.8	(ref)	(ref)
Once	1624	6.4	253	7.4	*1.19 (1.04–1.37)*	1.08 (0.93–1.25)
Twice	420	1.6	67	2.0	1.22 (0.92–1.59)	0.91 (0.69–1.22)
Three or more episodes	237	0.9	64	1.8	*2.07 (1.57–2.74)*	*1.50 (1.10–2.03)*
Pre-pregnancy antidepressant use						
3 months or less	5279	20.6	80	2.3	*0.26 (0.21–0.33)*	*0.27 (0.21–0.35)*
3 months–1 year	9790	38.2	563	16.4	(ref)	(ref)
1–2 years	4780	18.6	728	21.2	*2.65 (2.36–2.97)*	*2.61 (2.32–2.95)*
> 2 years	5812	22.6	2063	60.1	*6.17 (5.59–6.81)*	*6.10 (5.48–6.77)*

*AD* antidepressant medication, *CI* confidence interval, *EU* European Union, *OR* odds ratio. Significant OR marked in italics. ^a^F09–F95, except F10–16, F18–F31, F60.3, F90

## Discussion

In this large nationwide, population-based prospective cohort study, the majority of women receiving antidepressant medication in the year prior to conception discontinued treatment during the first half of the pregnancy. Among those who discontinued, nearly 12% re-initiated treatment during pregnancy. Women who were multiparous, who had higher education level, who were born in the Nordic countries or the EU (not including the Nordic countries), and with a more severe psychiatric history prior to pregnancy were more likely to re-initiate antidepressant treatment.

Previous studies show around 40–50% of women discontinue antidepressants, either shortly before or early in pregnancy (Charlton et al. 2015; Molenaar et al. 2020a), though there are large geographical differences in the prevalence of discontinuation or prescription rates (Charlton et al. 2015; Molenaar et al. 2020b). In the present study 3 in 4 women discontinued antidepressants during pregnancy. One previous study showed that due to concerns about adverse effects on the fetus, 75% of pregnant women discontinued SSRI treatment when planning a pregnancy, soon after discovering they are pregnant, or during the first trimester (Zoega et al. 2015). In line with a recent meta-analysis, the prevalence of antidepressant discontinuation decreased over the time period (Molenaar et al. 2020b).

In a small study of SSRI use among 132 women with depressive or anxiety disorders, more than half of women who discontinued treatment upon confirmation of pregnancy re-initiated treatment during pregnancy (Roca et al. 2013). In the present study, 11.8% of women re-initiated treatment during pregnancy. One recent report shows 17.6% of women who discontinued SSRIs during pregnancy restarted SSRIs postpartum (Molenaar et al. 2020a). Other studies have reported on the risk of depression relapse in women who discontinue antidepressant treatment during pregnancy, ranging from a five-fold increased risk (Cohen et al. 2006) to no increased risk (Molenaar et al. 2019). One study of pregnant women with recurrent depressive disorder found recurrence rates among women who continued antidepressant treatment to be similar to the rate in women who discontinued antidepressants (Yonkers et al. 2011). However, in this study, only one-fifth of women reported using antidepressants in the month preceding conception. We limited our study to women who had been dispensed an antidepressant drug in the year prior to pregnancy, and our findings are similar to those observed in a large cohort from the Netherlands (Ververs et al. 2006).

As hypothesized, it is not surprising that women with a more severe pre-pregnancy psychiatric history were more likely to re-initiate antidepressant treatment during pregnancy. Previous studies have shown longer duration of previous depression or antidepressant use and a greater number of previous depressive episodes to predict depression relapse during pregnancy (Cohen et al. 2006; Molenaar et al. 2019; Yonkers et al. 2011). Similarly, the strongest risk factor for recurrence of depression in the general population is the number of previous episodes (Hardeveld et al. 2010). Physicians caring for women with a greater pre-pregnancy mental health disease burden may also be more concerned about relapse risk, thus re-initiating antidepressants more promptly. In addition, women who were multiparous and women with higher education level were more likely to re-initiate treatment. Previous studies have shown higher parity to be associated with increased odds of continuation of antidepressants, whereas low socioeconomic status decrease odds of continuation (Molenaar et al. 2020a).

Key strengths of our study include its population-based design and the large sample size covering a 7-year period. Further, information on both antidepressant medications and psychiatric hospitalizations was obtained from national health data registers of high validity and level of detail, with mandatory reporting by health care professionals, as opposed to self-report. Previous studies have included highly selected samples from psychiatric specialty clinics (Cohen et al. 2006), carried out in hospital-based prenatal care settings (Molenaar et al. 2019; Yonkers et al. 2011), based on referrals from general practitioners or social media (Molenaar et al. 2019), or using Medicaid data from a wide range of treatment settings (Swanson et al. 2015).

One limitation of the study is residual confounding, in particular, severity of the depressive episode, co-morbidity between depression and anxiety, and a lack of information on variables not included in the registers that may have biased the observed associations. Moreover, we limited our study to women with antidepressant use in the year prior to pregnancy, and thus depressed women who discontinued antidepressants outside this time window were excluded and may represent another vulnerable group. In addition, we considered all antidepressants (ATC-code N06A), rather than specific drugs. It has been observed that relapse risk is higher following discontinuation of antidepressant drugs with greater effects on the serotonergic or noradrenergic system (Andrews et al. 2011). In addition, information on antidepressant use was based on dispensed prescriptions. Women’s adherence to antidepressant drugs dispensed is unknown. Whether women who discontinued antidepressant treatment during pregnancy were or stayed much less depressed than women who continued treatment is not known, and we lack knowledge of non-medical psychological treatment. Notably, the definition of relapse differs between the clinical and registry-based studies, with our study addressing re-initiation of antidepressant treatment during pregnancy which cannot necessarily be equaled with depression relapse. As we have no information on clinical relapse from structured psychiatric interviews or questionnaires, we are unable to determine the specific reasons for re-initiating medications in this population-based cohort. Further, as women were pregnant, many may have been reluctant to re-initiate treatment for the same reasons that prompted them to discontinue at the beginning of pregnancy. Based on our limited clinical information, the depression relapse rate is likely to have been substantially higher than that based only on re-initiated antidepressant treatment.

In conclusion, the majority of women discontinue antidepressant treatment during pregnancy with a small proportion of women re-initiating antidepressants during pregnancy. Pre-pregnancy psychiatric history appears particularly important for weighing the risks and benefits of discontinuing antidepressants. Better evidence beyond overall recurrence risk is needed to guide individual decisions (Berwian et al. 2017). Importantly, depression during pregnancy must not go untreated, and that psychological therapies are available where needed and that any discontinuation of antidepressant treatment is carried out in a controlled manner. Our results reinforce the previous finding that a greater burden of prior psychiatric illness is an important risk factor that should be considered by clinicians and patients, thus highlighting a need to closely monitor pregnant women with such a history.
